# *Artemisia argyi*-enhanced Mesenchymal Stem Cell Exosomes Alleviates Inflammation in C28/I2 Chondrocytes by inhibiting NF-κB

**DOI:** 10.7150/ijms.126119

**Published:** 2026-02-18

**Authors:** Shih-Wen Kao, Yu-Ting Hsu, Wei-Wen Kuo, Tai-Lung Huang, Kuan-Ho Lin, Chia-Hua Kuo, Dennis Jine-Yuan Hsieh, Tsung-Jung Ho, Shinn-Zong Lin, Chih-Yang Huang

**Affiliations:** 1 Graduate Institute of Aging Medicine, China Medical University, Taichung, Taiwan, ROC.; 2 Department of Orthopedic Surgery, Chung Shan Medical University Hospital, Taichung, Taiwan, ROC.; 3 School of Medicine, Chung Shan Medical University, Taichung, Taiwan, ROC.; 4 Cardiovascular and Mitochondrial Related Disease Research Center, Hualien Tzu Chi Hospital, Buddhist Tzu Chi Medical Foundation, Hualien, Taiwan, ROC.; 5 Department of Biological Science and Technology, College of Life Sciences, China Medical University, Taichung, Taiwan, ROC.; 6 School of Pharmacy, China Medical University, Taichung, Taiwan, ROC.; 7 Department of Orthopedics, Chung-Kang Branch, Cheng Ching General Hospital, Taichung, Taiwan, ROC.; 8 Department of Emergency Medicine, China Medical University Hospital, Taichung, Taiwan, ROC.; 9 College of Medicine, China Medical University, Taichung, Taiwan, ROC.; 10 Laboratory of Exercise Biochemistry, The Education University of Hong Kong, New Territories, Hong Kong.; 11 Soochow University, School of Physical Education and Sports Science, Suzhou, China.; 12 Department of Movement Sciences and Sports Training, School of sport sciences, University of Jordan, Jordan.; 13 Department of Medical Laboratory and Biotechnology, Chung Shan Medical University, Taichung, Taiwan, ROC.; 14 Clinical Laboratory, Chung Shan Medical University Hospital, Taichung, Taiwan, ROC.; 15 Department of Chinese Medicine, Hualien Tzu Chi Hospital, Buddhist Tzu Chi Medical Foundation, Tzu Chi University, Hualien, Taiwan, ROC.; 16 Integration Center of Traditional Chinese and Modern Medicine, Hualien Tzu Chi Hospital, Buddhist Tzu Chi Medical Foundation, Hualien, Taiwan, ROC.; 17 Department of Neurosurgery, Hualien Tzu Chi Hospital, Buddhist Tzu Chi Medical Foundation, Hualien, Taiwan, ROC.; 18 Bioinnovation Center, Buddhist Tzu Chi Medical Foundation, Hualien, Taiwan, ROC.; 19 Graduate Institute of Biomedical Sciences, China Medical University, Taichung, Taiwan, ROC.; 20 Department of Medical Laboratory Science and Biotechnology, Asia University, Taichung, Taiwan, ROC.; 21 Department of Medical Research, China Medical University Hospital, China Medical University, Taichung, ROC.

**Keywords:** osteoarthritis (OA), mesenchymal stem cells (MSC), exosomes, *Artemisia argyi*, NF-κB

## Abstract

Osteoarthritis (OA) is a degenerative joint disease with chronic inflammation, causing joint damage and function loss. Current treatments relieve symptoms but don't halt disease progression, highlighting the need for new therapies. Research shows mesenchymal stem cells (MSCs) can repair joints and reduce inflammation, but direct MSC injection may cause immune rejection, making MSC-derived exosomes a promising alternative. *Artemisia argyi* (AA) has antioxidant, anti-inflammatory, and anti-ageing effects that enhance stem cell function and cellular stability, making it a potential therapy for OA. This study explores whether AA extract can enhance exosomes from Wharton's jelly stem cells (WJSCs) for OA treatment. Results revealed that AA promoted WJSCs proliferation and increased both the yield and size of exosomes. Furthermore, AA-enhanced exosomes significantly suppressed NF-κB pathway related proteins (p-IKKα/β and p-NF-κB) and the matrix degrading protein MMP13 while increasing the expression of the cartilage extracellular matrix protein COL2A1, resulting in a greater reduction of NF-κB signaling proteins and MMP13 expression, along with increased COL2A1 levels. Additionally, these exosomes effectively reversed H₂O₂-induced ROS accumulation, with antioxidant effects surpassing those of untreated exosomes. Further studies using NF-κB activators confirmed that the therapeutic effects of these exosomes were primarily mediated by inhibition of the NF-κB signalling pathway. In conclusion, AA-enhanced WJSCs exosomes improved the proliferation, anti-inflammatory, and antioxidant capacities of C28/I2 chondrocytes under oxidative stress. These findings highlight their potential to reduce ROS levels, regulate pro-inflammatory proteins, and inhibit the NF-κB pathway, offering a promising strategy for protecting cartilage against damage caused by inflammatory joint diseases.

## Introduction

Osteoarthritis (OA) is a chronic degenerative joint disease primarily characterized by the progressive degradation of articular cartilage and structural changes in surrounding joint components, predominantly affecting older adults 1. OA not only leads to chronic pain and joint stiffness but also significantly impairs daily activities, reducing the quality of life and causing psychological stress in many patients. Osteoarthritis has five stages, from stage 0 (normal knee) to stage 4 (severe degeneration). Stages 1 and 2 involve mild symptoms, while stage 3 shows significant cartilage damage and pain. Stage 4 is the most severe, with nearly complete cartilage loss and functional impairment. Diagnosis is typically confirmed through X-ray imaging, revealing joint space narrowing, cartilage loss, and osteophyte formation 2.

The pathogenesis of OA involves a complex interplay of mechanical, genetic, and biochemical factors 3. Mechanical stress from repetitive joint use or injury can accelerate cartilage damage, while genetic factors, such as GDF5 gene mutations 4, have been linked to increased susceptibility to cartilage degradation. At the molecular level, the NF-κB signalling pathway plays a central role in OA progression. Under normal conditions, NF-κB is bound to the inhibitor IκB in the cytoplasm. Upon activation by inflammatory stimuli, IκB is degraded, allowing NF-κB to translocate to the nucleus and promote the transcription of pro-inflammatory cytokines, such as TNF-α, IL-1β, and IL-6, which contribute to joint inflammation, chondrocyte apoptosis, and cartilage matrix breakdown 5-7. Additionally, NF-κB activation upregulates matrix metalloproteinases (MMPs), particularly MMP-3 and MMP-13, which degrade cartilage components like collagen and aggrecan, exacerbating joint destruction 8, 9. Persistent NF-κB activation and its interaction with pathways such as SDF-1/CXCR4 10 further accelerate cartilage degradation through PI3K/Akt signalling and TGF-β/Smad3 axis dysregulation 11, 12.

Despite advancements in understanding OA pathogenesis, current clinical treatments remain largely symptomatic, aimed at reducing pain and maintaining joint function. For mild pain, analgesics like acetaminophen or Non-Steroidal Anti-Inflammatory Drugs (NSAIDs) are commonly prescribed 13, 14. These medications, however, do not reverse joint damage and often lead to adverse side effects, including gastrointestinal ulcers, cardiovascular risks, and kidney damage with long-term use 15. Corticosteroid injections provide short-term relief but require periodic repetition, while surgical interventions, such as total knee replacement, carry risks like infection, blood clots, and prolonged recovery periods 16. Moreover, clinical treatment does not effectively target the underlying causes of cartilage degradation, such as inflammatory cytokines and matrix metalloproteinases (MMPs), which drive disease progression. These limitations highlight the urgent need for novel therapeutic approaches targeting the underlying mechanisms of OA.

Recent advances in regenerative medicine have highlighted the therapeutic potential of mesenchymal stem cells (MSCs) in treating OA. MSCs possess self-renewal and multipotent differentiation capabilities, allowing them to repair damaged tissues such as cartilage, bone, and ligaments. Clinical studies have shown that MSC injections can reduce pain and improve joint function in OA patients. For instance, MSC therapy in knee OA patients has demonstrated sustained pain relief for 6-12 months and an increase in cartilage thickness 17. Additionally, *in vitro* studies reveal that MSCs inhibit the NF-κB pathway, thereby reducing inflammation and apoptosis in chondrocytes. MSCs exert anti-inflammatory and immunomodulatory effects, which further promote joint repair and regeneration 18.

Traditional Chinese Medicine (TCM) has also gained attention for its potential role in enhancing the efficacy of regenerative therapies, including MSC-derived exosome treatments. Exosomes, nano-sized vesicles secreted by MSCs, carry bioactive molecules like proteins, lipids, and nucleic acids that mediate cell-to-cell communication and promote tissue repair. MSC-derived exosomes reduce pro-inflammatory cytokines (e.g., TNF-α, IL-1β) and enhance extracellular matrix production, facilitating cartilage regeneration 19. Research has shown that certain TCM herbs, such as Astragalus and Ginseng, can promote MSC proliferation and differentiation while enhancing exosome secretion. For example, when MSCs are cultured with Astragalus extracts, exosome production is significantly enhanced, carrying higher levels of anti-inflammatory factors like TGF-β and VEGF 20, 21. Similarly, preclinical studies have indicated that MSC-derived exosomes preconditioned with TCM herbs show enhanced cartilage repair in animal OA models. Similarly, *Artemisia argyi* (AA), a key herb in TCM, has been found to promote MSC proliferation, exosome secretion 22, chondrogenic differentiation, and enhancing the regenerative potential of MSCs, particularly for OA treatment 23. Although several TCM herbs, such as ginseng and astragalus, have been reported to enhance mesenchymal stem cell proliferation and exosome secretion, these botanicals primarily exert immunomodulatory and pro-survival effects. In contrast, *A. argyi* possesses a distinct phytochemical profile enriched in lipophilic flavonoids and sesquiterpenes, including eupatilin and nerolidol, which exhibit potent antioxidant, mitochondrial-protective, and NF-κB regulatory activities. Given that oxidative stress driven NF-κB activation is a central pathological mechanism in osteoarthritis, A. argyi represents a rational and disease-oriented choice for stem cell preconditioning. Moreover, emerging evidence suggests that *A. argyi* modulates stem cell senescence and metabolic fitness, indicating a mode of functional enhancement that differs mechanistically from previously studied herbal modulators.

These developments not only highlight the synergy between modern regenerative medicine and herbal knowledge but also pave the way for novel therapeutic approaches that combine the best of both worlds. This paper will explore the mechanisms underlying OA progression and current treatment challenges, focusing on the regenerative potential of MSCs and exosomes. Furthermore, it will examine how TCM, particularly AA, can enhance exosome-based therapies, providing insights into innovative strategies for OA management.

## Materials and Methods

### Antibodies and reagents

The following antibodies were used in the current study: CD9 (#13174; Cell signaling technology, Danvers, MA, USA), CD63(Merck Millipore; Burlington, MA, USA), CD81(sc-166028; Santa Cruz Biotechnology, Dallas, TX, USA), Calnexin (#2679, Cell signaling), β-actin(sc-47778, Santa Cruz), p-IKKα/β (bs-3237R; Bioss Antibodies, Woburn, MA, USA), p-NF-κB p-p65 (#3033, Cell signaling), NF-κB p65(#8242, Cell signaling), p-IκB-α (#2859, Cell signaling), IκB-α (sc-1643, Santa Cruz), COL2A1 (GB11021; Servicebio, Wuhan, Hubei, China), MMP-13 (GTX100665; GeneTex, Irvine, CA, USA), Nanog (#4903, Cell Signaling), KLF4 (#4038, Cell signaling), CXCR4 (60042-1-Ig; Proteintech, San Diego, CA, USA), CXCR7 (bs-4897R, Bioss), CD44 (#3570, Cell signaling), CD90 (sc-53456, Santa Cruz). Chemicals/reagents included: 3-(4,5-dimethyl-2-thiazolyl)-2,5-diphenyl-2H-tetrazolium bromide, MTT reagent, H_2_O_2_, HY-134476 (NF-κΒ activator 1) were from MedChemExpress, CM-H_2_DCFDA (ab113851; Abcam, Waltham, MA, USA), ProLong Diamond Antifade Mountant with DAPI and Phalloidin (B7474; Thermo Fisher Scientific; Waltham, MA, USA), and PageRuler Prestained Protein Ladder (Thermo Fisher Scientific) ExoSparkler Exosome Membrane Labeling Kit-Green (EX02; Dojindo, Munich, Germany). All other chemicals utilized were of the highest grade commercially available and acquired from either Sigma-Aldrich or Thermo Fisher Scientific.

### *Artemisia argyi* water extract preparation

Dried *Artemisia argyi* leaves were obtained through a procurement process from Hongwei Company in Huizhou, Guangdong, China. A total of 300 g of these leaves were boiled in 3 liters of double-distilled water until the extract volume was reduced to 500 ml. The crude extract was then centrifuged at 10,000 rpm for 15 mins at 4°C, and the supernatant was carefully removed using a dropper. This supernatant was subjected to a secondary filtration process to eliminate any remaining debris. The resulting clarified *Artemisia argyi* aqueous extract (AA) was measured to have a concentration of 20 mg/ml and was subsequently stored at -20°C for future use.

### Cell culture

The human Wharton's jelly-derived mesenchymal stem cells (WJSCs, Bioresource Collection and Research Center, RM60596) were cultured in mesenchymal stem cell medium (MSCM, ScienCell Research Laboratories, catalog number #7501), supplemented with mesenchymal stem cell growth supplement (MSCGS, ScienCell Research Laboratories, catalog number #7552), 10% charcoal-treated fetal bovine serum (FBS), and 1% PS (100 units/mL penicillin and 100 μg/mL streptomycin), maintained in a 37°C, 5% CO₂ incubator.

The C28/I2 human chondrocyte cell line was selected as an *in vitro* model because it is a well-characterized and widely used system for studying chondrocyte inflammatory and oxidative stress responses. Compared with primary human chondrocytes, C28/I2 cells offer improved experimental reproducibility and reduced donor variability, facilitating mechanistic evaluation of NF-κB signaling and exosome-mediated effects under controlled conditions. The C28/I2 cell line, derived from human embryonic chondrocytes, was purchased from Merck Millipore (catalog number SCC043) and cultured in DMEM High Glucose medium containing 10% FBS and penicillin-streptomycin (100 units/mL penicillin and 100 μg/mL streptomycin), similarly maintained in a 37°C, 5% CO₂ incubator. Once the cells reached the required confluence for experimentation, the chondrocytes were treated with 100 μM H₂O₂ for 2 hours, followed by various treatments (such as conditioned medium, exosomes, and chemical reagents) according to the experimental design.

### Exosome isolation and identification

WJSCs were seeded at a density of 6 × 10⁵ cells per plate in 150 mm culture dishes. After incubation for 24 hours, the cells were treated with AA for 4 hours. Following the AA treatment, the medium was refreshed and cultured for 20 hours under conditions without fetal bovine serum (FBS). Subsequently, the conditioned medium was harvested and filtered using a 0.2 μm filter to remove cell debris and other impurities. The filtered medium was then transferred to a Vivaspin 30 kDa concentrator (Cytiva, 45-001-580), concentrating the original 80 ml of conditioned medium to 5 ml for subsequent exosome extraction. To precipitate the exosomes, 1 ml of ExoQuick-TC (SBI, EXOTC50A-1) was added to the concentrated medium and incubated at 4°C for 24 hours to promote exosome precipitation. After incubation, exosomes were obtained in the form of a pellet through centrifugation, and the pellet was then dissolved in PBS for further experimental analysis.

### Cell viability assay

Cell viability was assessed using the MTT assay (3-[4,5-dimethylthiazolyl-2]-2,5-diphenyltetrazolium bromide), which measures mitochondrial succinate dehydrogenase activity by detecting the metabolic reduction of MTT into purple formazan crystals. WJMSCs and C28/I2 cells were seeded in appropriate 96-well plates (1×10³ cells/well for WJMSCs and 1×10⁴ cells/well for C28/I2) and cultured overnight at 37°C in a humidified incubator with 5% CO₂. Following various experimental treatments, including exposure to *Artemisia argyi* water extract (10-300 μg/mL) for 24 hours or H₂O₂ (50-300 μM) for 2 hours, MTT solution (0.5 mg/mL) was added and incubated at 37°C for 4 hours. The resulting formazan crystals were dissolved in dimethyl sulfoxide (DMSO), and absorbance was measured at 570 nm using a microplate reader or spectrophotometer. Cell viability was expressed as a percentage relative to the untreated control (100%).

### Western blot analysis

Cells were scraped and washed twice with PBS, followed by preparation of cell lysates and protein extraction using RIPA (radioimmunoprecipitation assay) lysis buffer (Thermo Fisher Scientific) supplemented with protease and phosphatase inhibitors. The cell pellets were lysed on ice for 30 mins at 4°C, then centrifuged at 12,000 rpm for 40 mins at 4°C. The supernatant was collected and stored at -20°C for future use. Mitochondrial and cytosolic protein fractions were isolated using a commercially available reagent (Mitochondria Isolation Kit, Thermo Fisher Scientific) as previously described 24. Equal amounts of protein (20 μg/well) were loaded onto the SDS-PAGE gel and separated at 60 volts for 30 mins in the stacking gel and 80 volts for 100 mins in the resolving gel. The proteins were then transferred to a 0.45 μm polyvinylidene difluoride (PVDF) membrane (Sigma-Aldrich) at 100 volts for 90 mins. After transfer, the membrane was blocked with 10% skim milk in Tris-buffered saline with 0.1% Tween® 20 (TBST) at 25^o^C for 1 hour. It was then incubated with the primary antibody overnight at 4°C. Following incubation with the secondary antibody and the appropriate host species at 25^o^C for 1 hour, the blot was detected using the Fuji LAS 3000 imaging system. As previously described, band intensities were measured using ImageJ software (NIH, Bethesda, Maryland, USA)^25^.

### H_2_DCFDA (2′,7′-Dichlorodihydrofluorescein Diacetate) staining

Intracellular ROS generation in C28/I2 cells was assessed using the cell-permeable cationic reagent CM-H_2_DCFDA, following the manufacturer's instructions. Cells were cultured in a 4-chamber slide at densities of 1.2×10⁵ cells/well for C28/I2. After treatment, the cells were incubated with 10 μM H_2_DCFDA in the dark for 45 mins at 37^o^C, followed by a single wash with PBS. ROS production was determined as previously described 26, by measuring DCF fluorescence (green) intensity using a fluorescence microscope to assess intracellular ROS levels.

### Reverse transcription-quantitative polymerase chain reaction (RT-qPCR) assay

Human chondrocytes, following various treatments, underwent RNA extraction using Trizol (15596026, Thermo Fisher Scientific) in 60 mm culture dishes. RNA concentration and purity were assessed using a microspectrophotometer. Reverse transcription was carried out with the Mir-X miRNA First-Strand Synthesis Kit (#638313; Takarabio, San Jose, CA, USA). RT-qPCR was then performed using iQ™ SYBR® Green Supermix (#1708880; Bio-Rad, Hercules, CA, USA) on the QuantStudio™ 3 Real-Time PCR System. Primers were designed using NCBI Primer Blast and synthesized by Invitrogen. Data analysis was conducted using the 2-ΔΔCt method, with normalization to the endogenous control GAPDH. Table 1 below provides the primer sequences used in the study.

### Immunofluorescence (IF) staining

C28/I2 cells (1.2×10⁵ per well) were grown in a 4-chamber slide and fixed with 4% paraformaldehyde (PFA) for 15 mins following the designated treatments. Permeabilization was performed using 0.1% Triton X-100 in PBS for 15 mins. Blocking was carried out with goat serum in PBS for 1 hour at room temperature. Cells were then incubated with the primary antibody (NF-κB p65, #8242) in 1% BSA in PBST at 4°C overnight. Subsequently, secondary antibodies, specifically Alexa Fluor® 488 goat anti-mouse IgG (H+L) *highly cross-adsorbed (A11001), were applied, and fluorescence levels were evaluated using fluorescent microscopy.

### Nuclear and cytoplasmic fractionation

After collecting cells by centrifugation at 600 x g for 5 mins at 4°C, the cells were resuspended in 0.2 mL of Extraction Buffer VI/CEB-A Mix containing DTT II/DTT and protease inhibitors. The suspension was vortexed vigorously at the highest setting for 15 seconds and incubated on ice for 10 mins. Next, 11 μL of ice-cold Lysis X/Cytosol Extraction Buffer-B was added, followed by vortexing for 5 seconds and 1 minute of incubation on ice. The mixture was then centrifuged at 16,000 x g for 5 mins. The resulting supernatant (containing cytoplasmic extracts) was transferred to a clean, pre-chilled tube. The pellet was resuspended in 100 μL of ice-cold Nuclear Extraction Buffer Mix, vortexed for 15 seconds, and incubated on ice for 10 mins. This process was repeated for a total of 40 mins. Finally, the sample was centrifuged at 16,000 x g for 10 mins to collect the supernatant, which contains the nuclear extracts, and immediately transferred to a new pre-chilled tube. The sample was stored at -80°C.

### Statistical analysis

All experiments were performed in triplicates and analyzed using one-way analysis of variance (ANOVA), followed by Tukey's multiple comparison test to determine significant differences between groups. Statistical analysis was conducted using GraphPad Prism software (San Diego, CA, USA), and all measurement data are expressed as mean ± standard deviation. Group differences were assessed through ANOVA, with p < 0.05 considered statistically significant.

## Results

### AA treatment enhances WJSC proliferation, maintains stem cell characteristics, and significantly optimizes exosome production

**Figure 1:**
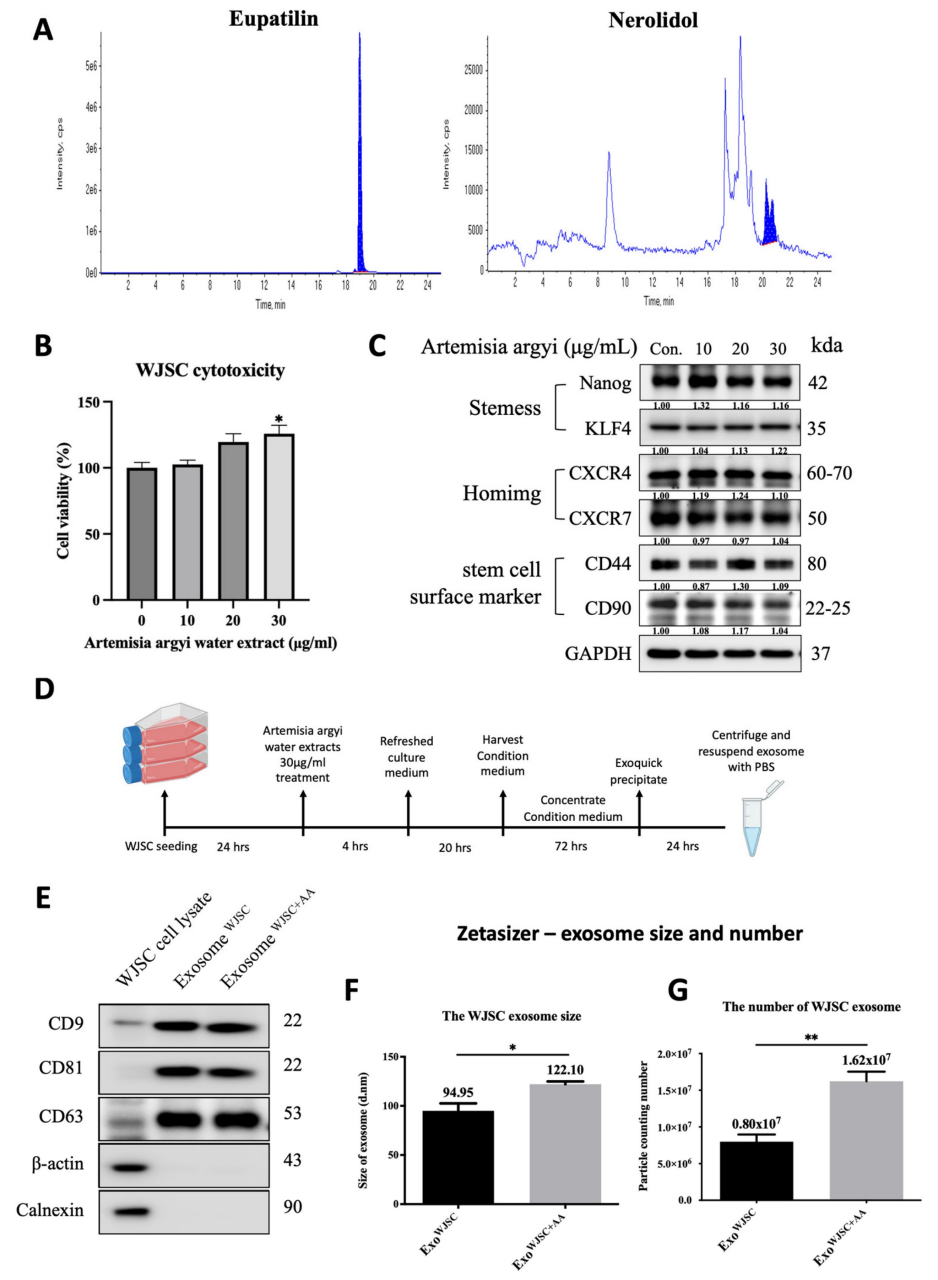
** AA Enhances exosome size and significantly increases exosome yield.** (A) Chromatogram of crude extract of *Artemisia argyi* showing major peaks corresponding to Eupatilin and Nerolidol, analyzed by (AB Sciex Instruments QTRAP 5500). (B) the MTT assay demonstrates a dose-dependent effect of AA treatment (0-30 μg/ml) on WJSCs viability, with higher concentrations leading to progressively enhanced cell survival after 24 hours. (C) western blot analysis reveals that AA treatment modulates the expression of key markers, including stemness markers (Nanog and KLF4), homing markers (CXCR4 and CXCR7), and stem cell surface markers (CD44 and CD90), suggesting its regulatory role in cell survival and functional properties. (D) the schematic diagram illustrates the AA treatment protocol for WJSCs, where cells were treated with AA for 4 hours, followed by a medium change and incubation for an additional 22 hours before conditioned medium collection. Exosomes were isolated using Exoquick and confirmed through (E) Western blot analysis of specific markers (CD9, CD81, and CD63). (F, G) Characterization using a zeta-sizer revealed the size and number distribution of the exosomes. Data are presented as mean ± SD (n = 3). Statistical analysis (one-way ANOVA) showed significant differences *p < 0.05, **p < 0.01 vs. EXO^WJSC^, confirming the enhanced biological effects of AA-treated WJSCs and their exosomes.

The mass spectrometry analysis of *Artemisia argyi* crude extract identified Eupatilin and Nerolidol as the primary components, with the chromatogram (Figure 1A) showing a single dominant peak corresponding to these compounds. To determine the optimal concentration of AA for use in WJSCs, an MTT assay was performed. The results demonstrated that AA enhanced WJSCs viability and proliferation in a dose-dependent manner within the concentration range of 0 to 30 μg/mL. At 30 μg/mL, cell viability was significantly higher compared to the control group (Figure 1B). Additionally, AA treatment at concentrations up to 30 μg/mL did not significantly reduce WJSC viability, as assessed by MTT assay.

To further confirm whether AA treatment affects the stem cell characteristics of WJSCs, we performed Western blot analysis to examine the expression levels of various stem cell markers. The results showed that at an AA concentration of 30 μg/mL, there were no significant changes in the expression levels of stem cell markers (such as Nanog Homeobox and Nanog; Kruppel-Like Factor 4, KLF4), homing markers (C-X-C chemokine receptor type 4, CXCR4; C-X-C chemokine receptor type 7, CXCR7), and stem cell surface markers (cluster of differentiation 44, CD44; cluster of differentiation 90, CD90) (Figure 1C). Based on these findings, we selected an AA concentration of 30 μg/mL to treat WJSCs for subsequent experiments.

In follow-up experiments, WJSCs were treated with 30 μg/mL AA, after which the conditioned medium and secreted exosomes were collected (Figure 1D). Following a standardized isolation procedure, exosome-specific markers CD9, CD81, and CD63 were successfully verified by western blot (Figure 1E), ensuring high-quality exosome isolation. Additionally, Zetasizer analysis showed that AA treatment not only increased exosome size (Figure 1F) but also significantly enlarged exosome yield (Figure 1G). AA treatment increased exosome yield, as indicated by a higher particle number and a modest shift in vesicle size distribution compared with untreated WJSC-derived exosomes.

Although AA treatment did not induce cytotoxicity across the tested concentration range, 30 μg/mL was selected for subsequent experiments because it produced the most robust enhancement in WJSC proliferation while preserving stem cell identity. Importantly, this concentration did not alter the expression of key stemness-related transcription factors (Nanog and KLF4), homing-associated receptors (CXCR4 and CXCR7), or surface markers (CD44 and CD90), indicating that AA-induced proliferative activation occurred without promoting premature differentiation. Given that exosome biogenesis is closely linked to cellular metabolic and proliferative activity, the enhanced proliferation observed at 30 μg/mL AA provides a functional basis for optimizing exosome yield and downstream therapeutic efficacy.

### AA-enhanced WJSC-derived exosomes exhibit superior anti-inflammatory and chondroprotective effects

**Figure 2:**
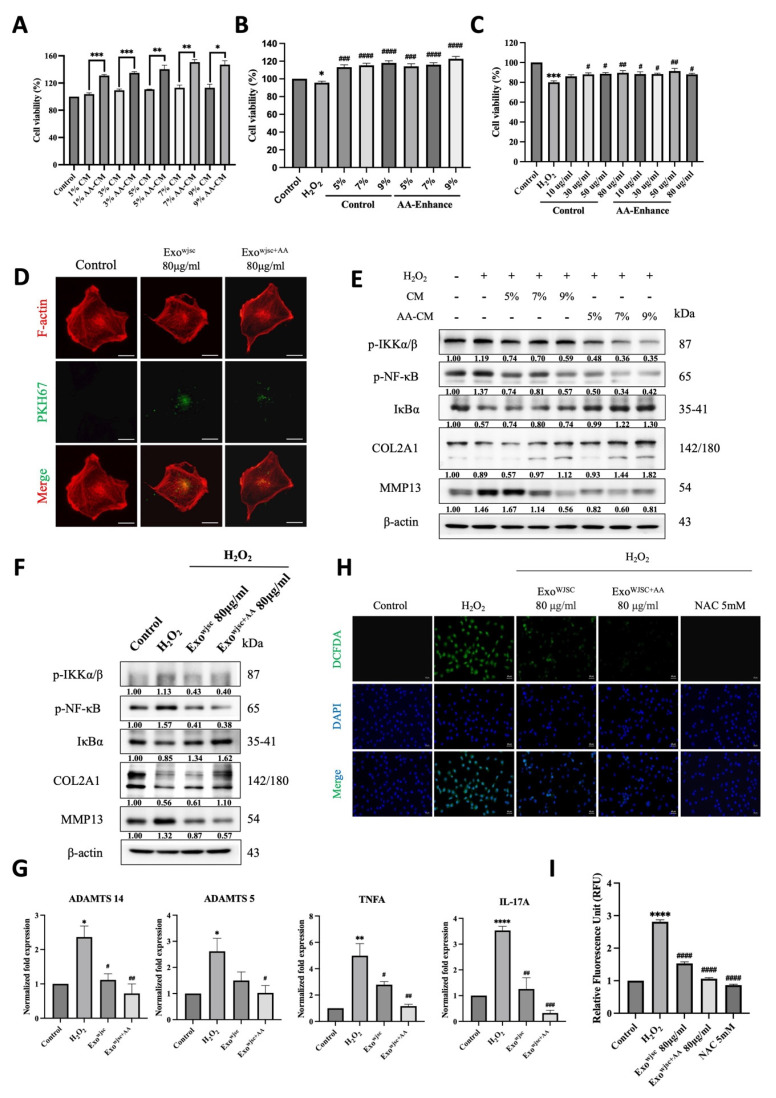
** C28/I2 cells were exposed to H₂O₂ (100 μM, 2 h) followed by treatment with AA-enhanced WJSC-conditioned medium or exosomes. Cell viability, exosome uptake, NF-κB signaling proteins, cartilage-associated markers, and intracellular ROS levels were analyzed.** (A) AA-enhanced conditioned medium at concentrations of 1%, 3%, 5%, 7%, and 9% for 24 hours significantly promoted C28/I2 cell proliferation compared to the control group. (B) After 2 hours of oxidative stress with H₂O₂, treatment with AA-enhanced conditioned medium restored cell viability in a dose-dependent manner. (C) Similarly, treatment with increasing concentrations of AA-enhanced WJSCs exosomes after H₂O₂ exposure enhanced cell viability. (D) Fluorescence microscopy confirmed cellular uptake of PKH67-labeled exosomes, with F-actin filaments stained in red (Scale bar = 20 μm). (E) Immunoblot analysis showed decreased expression of inflammation markers (p-IKKα/β, p-NF-κB, IκBα) and OA marker MMP13, with increased COL2A1 at higher concentrations of AA-enhanced conditioned medium (5%, 7%, 9%). (F) Similarly, AA-enhanced WJSCs exosomes (80 μg/mL) modulated inflammatory and OA markers. (G) mRNA expression of ADAMTS14, ADAMTS15, TNF-α, and IL-17A was normalized to β-actin using the 2-∆∆Ct method. (H) ROS generation was assessed by DCFDA staining. Fluorescence microscopy showed reduced ROS levels in cells treated with AA-enhanced exosomes (80 μg/mL) compared to the H₂O₂ group (Scale bar = 40 μm). (I) Quantification confirmed significant reduction in ROS levels, emphasizing the antioxidant effects of AA- conditioned vesicles. Data are presented as mean ± SD (n = 3), *p < 0.05, **p < 0.01, ***p < 0.001, ****p < 0.0001 vs. untreated control group; #*p* < 0.05, ##*p* < 0.01, ###*p* < 0.001, ####*p* < 0.0001 vs. H₂O₂ treated group.

Since exosomes are isolated from a conditioned medium, both the conditioned medium and exosomes were evaluated in the subsequent assessment for arthritis treatment. To investigate the effect of AA-enhanced conditioned medium on cell proliferation, C28/I2 chondrocytes were treated with various concentrations of conditioned medium (1%, 3%, 5%, 7%, and 9%) for 24 hours. The results (Figure 2A) indicate that cell proliferation significantly increased compared to untreated conditioned medium, suggesting that AA-enhanced conditioned medium promotes chondrocyte growth.

Furthermore, cell viability was evaluated by treating H₂O₂-damaged cells with conditioned medium (5%, 7%, and 9%) or exosomes (80 μg/mL). The findings showed that while cell viability decreased under H₂O₂-induced injury, it was significantly restored after treatment with a conditioned medium (Figure 2B) and exosomes (Figure 2C), highlighting their protective effects under oxidative stress. Additionally, the successful uptake of exosomes by chondrocytes was confirmed using PKH67-labeled exosomes (green fluorescence) and phalloidin-stained F-actin filaments (red fluorescence, marking the cytoplasmic region). As shown in (Figure 2D), exosomes were effectively internalized, supporting a potential mechanism for their protective effects on cells.

Western blot analysis was conducted to evaluate the expression of inflammation-related and OA markers (Figures 2E and 2F). The results showed that AA-enhanced WJSCs conditioned medium and exosomes significantly reduced the levels of NF-κB signaling pathway proteins (p-IKKα/β and p-NF-κB) and the OA marker matrix metalloproteinase 13 (MMP13), while increasing the expression of the cartilage marker collagen type II alpha 1 (COL2A1), demonstrating anti-inflammatory properties and stabilization of the chondrocyte matrix under oxidative stress. Consistently, H₂O₂ treatment significantly upregulated inflammatory markers (e.g., TNF-α and IL-17A) and cartilage matrix degradation markers (e.g., ADAMTS14 and ADAMTS5). However, WJSCs-derived exosomes markedly reduced the expression levels of these genes (Figure 2G), indicating their role in promoting cartilage repair and inhibiting excessive bone formation. Notably, exosomes derived from AA-treated WJSCs produced a statistically greater reduction in ROS levels and NF-κB activation compared with exosomes derived from untreated WJSCs in this *in vitro* model, further highlighting their greater potential in enhancing cartilage repair and restoration.

ROS production was assessed using DCFDA staining and fluorescence microscopy. As shown in (Figures 2H and 2I), cells treated with AA-enhanced WJSCs exosomes exhibited a significant reduction in ROS production compared to those treated with standard WJSCs exosomes, reaching levels comparable to cells treated with the known ROS inhibitor NAC (5 mM). These results indicate that AA-enhanced WJSCs exosomes are highly effective in reducing oxidative stress.

### AA-Enhanced WJSC-derived exosomes protect cartilage by inhibiting the NF-κB inflammatory pathway

**Figure 3:**
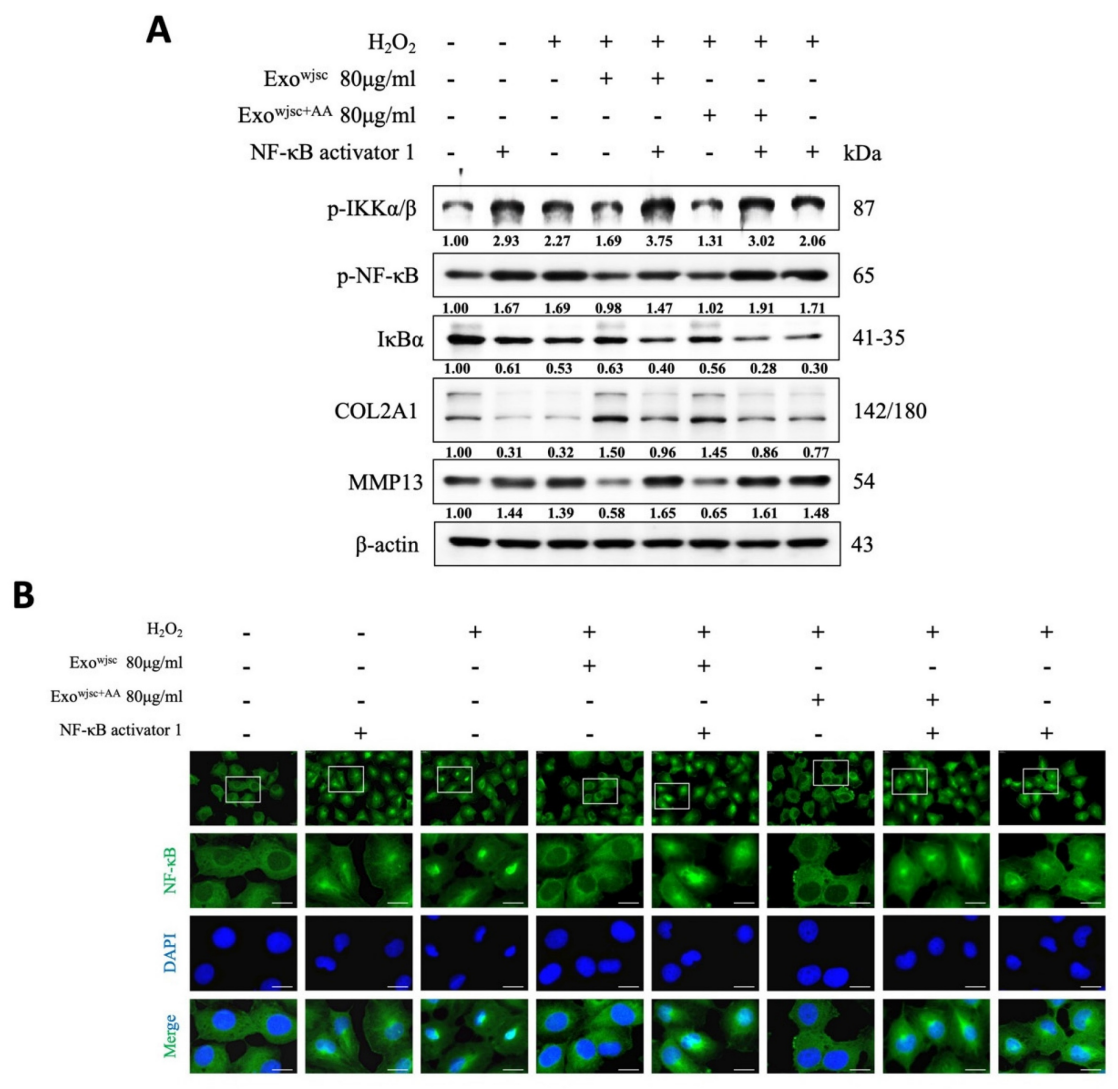
** Verification of AA-enhanced exosomes reducing H₂O₂-induced inflammation in C28/I2 cells and supporting cartilage homeostasis via inhibition of the NF-κB pathway using an NF-κB activator.** C28/I2 cells were pretreated with H_2_O_2_ for 2 hours and then co-treated with standard WJSCs exosomes or AA-enhanced WJSCs exosomes (80 μg/mL) with NF-κB activator (5μM) for 22 hours. (A) Western blot analysis reveals expression levels of key inflammation-related proteins, including p-IKKα/β, p-NF-κB, and IκBα, as well as OA markers MMP13 and COL2A1. (B) Translocation of p65 was determined using a NF-κB p65 antibody and an Alexa Fluor 488-conjugated anti-rabbit IgG antibody. Nuclei were counterstained with DAPI. Scale bar = 40 μm.

To confirm that these exosomes can reduce H₂O₂-induced inflammation in C28/I2 cells and support cartilage homeostasis through inhibition of the NF-κB pathway, we conducted further experiments using an NF-κB activator. As shown in Figure 3A consistent with previous findings, treatment with both standard WJSCs exosomes and AA-enhanced WJSCs exosomes under H₂O₂ conditions significantly reduced the expression of NF-κB signaling-related proteins and the OA marker MMP13, while increasing the expression of the cartilage marker COL2A1. However, these effects were reversed after treatment with the NF-κB activator, further confirming that the exosomes exert their therapeutic effects by inhibiting the NF-κB pathway.

Subsequently, we used immunofluorescence staining to localize the distribution of NF-κB in the cells (Figure 3B). The results showed that under treatment with either the NF-κB activator or H₂O₂ induction, NF-κB significantly translocated to the nucleus. However, following treatment with both standard WJSCs exosomes and AA-enhanced WJSCs exosomes, the green fluorescence intensity in the cells was notably reduced, indicating that NF-κB nuclear translocation was effectively suppressed. Finally, these effects were reversed after subsequent treatment with the NF-κB activator, further confirming that exosomes exert their therapeutic effects by inhibiting the NF-κB pathway.

## Discussion

This study explores the potential of AA to enhance WJSCs function and its impact on exosome production. The findings provide crucial insights into how AA treatment affects stem cell characteristics and exosome generation. To ensure that the exosomes used in this study were secreted by WJSCs, we specifically examined the expression of stem cell-related proteins, such as Nanog, and KLF4, which are essential transcription factors for maintaining stem cell self-renewal and pluripotency. If the expression of these markers is inhibited, stem cells are more likely to enter a differentiation state, making it difficult to ascertain whether the collected exosomes originated from undifferentiated stem cells or differentiated cells. Further experiments confirmed the increased yield and quality of exosomes derived from AA-treated WJSCs. Western blot analysis showed the presence of exosome-specific markers (such as CD9, CD81, CD63), verifying the reliability of the exosome isolation method 27. Notably, AA treatment not only increased the exosome yield but also significantly altered their size, suggesting that AA may have a potential regulatory effect on the biogenesis of exosomes. Previous studies have shown that increasing exosome production could enhance their clinical applicability, providing theoretical support for the use of AA-enhanced exosomes in clinical applications 28, 29.

NF-κB plays a critical regulatory role in the progression of OA, with its activation promoting inflammation, cartilage degradation, and joint structural damage. In OA, chondrocytes activate the NF-κB signaling pathway, releasing large amounts of pro-inflammatory and catabolic cytokines and chemokines, such as TNF-α, IL-1β, IL-6, IL-8, and RANKL. These factors stimulate the secretion of matrix metalloproteinases (MMPs), including MMP-2, MMP-9, and MMP-13 30, 31, while reducing the production of COL2A1 and proteoglycans, leading to cartilage matrix degradation. Moreover, the sustained activation of NF-κB creates a positive feedback loop that further amplifies its activity and promotes the activation of other regulatory transcription factors, such as RUNX2 and HIF-2α. These transcription factors enhance the overexpression of MMP-13 and ADAMTS-5 32, driving chondrocytes from a pre-hypertrophic stage to terminal hypertrophy, accelerating cartilage tissue degeneration and destruction 33, 34. In our study, we found that AA-enhanced culture media and exosomes significantly inhibited NF-κB signaling activity, effectively reducing MMP-13 expression while promoting COL2A1 protein synthesis. qPCR data showed that these exosomes were more effective in reversing the excessive production of pro-inflammatory cytokines (such as TNF-α and IL-17A) induced by H₂O₂ in C28/I2 chondrocytes and in suppressing the overexpression of ADAMTS-12 and ADAMTS-5. Further experiments using NF-κB activators confirmed that the inhibition of NF-κB signaling by AA- conditioned vesicles was reversed upon activation of NF-κB. Additionally, immunofluorescence imaging revealed that the previously observed inhibition of NF-κB nuclear translocation by these exosomes was also reversed upon administration of the NF-κB activator. These findings strongly support that the primary mechanism of action of AA-enhanced exosomes is the inhibition of the NF-κB signaling pathway.

Excessive reactive oxygen species (ROS) generation plays a key role in OA progression, with H₂O₂ serving as a major ROS component and oxidative stress marker 35-37. Elevated H₂O₂ levels induce cell damage, matrix degradation, and inflammation, activating pathways like NF-κB and MAPK. This stimulation promotes chondrocytes to secrete proinflammatory cytokines (e.g., TNF-α, IL-1β) and MMPs, driving OA-related inflammatory and catabolic changes 38, 39. Given the central role of oxidative stress in OA pathogenesis, targeting ROS represents a promising therapeutic approach. In this study, AA-enhanced WJSCs exosomes were evaluated for their antioxidant effects. DCFDA staining revealed that these exosomes significantly reduced H₂O₂-induced ROS production in C28/I2 chondrocytes, demonstrating their potential to alleviate oxidative stress and support their therapeutic application in inflammatory joint diseases.

The mass spectrometry analysis of this study showed that the primary components of *Artemisia argyi* crude extract are Eupatilin and Nerolidol, which have been confirmed in previous studies to have significant therapeutic effects on arthritis. While both compounds are well documented for their anti-inflammatory, antioxidant, and cytoprotective properties, the present study did not directly determine whether these phytochemicals are incorporated into WJSC-derived exosomes. Eupatilin alleviates OA pain and protects cartilage by reducing oxidative stress, inhibiting catabolic enzymes (such as MMP-13 and ADAMTS-5), and modulating the JNK pathway, which is consistent with the anti-inflammatory effects of exosomes 23, 40, 41. Nerolidol significantly reduces the expression of pro-inflammatory cytokines, the activity of inflammatory enzymes, and enhances antioxidant enzyme activity 42, 43, demonstrating strong anti-inflammatory potential, further supporting the inhibitory effect of exosomes on the NF-κB pathway in this study. Mesenchymal stem cell (MSC)-derived exosomes show significant potential in OA treatment, promoting MSC migration and cartilage repair. In one study, exosomes derived from human bone marrow mesenchymal stem cells (hBMSCs) were taken up by chondrocytes, significantly enhancing cell activity under IL-1β treatment and upregulating genes such as Survivin, Aggrecan, and SOX9, while inhibiting the expression of pro-inflammatory genes like NF-κB. Additionally, exosomes restored the expression of TGF-β, demonstrating protective effects by inhibiting apoptosis and inflammation 44, 45. Eupatilin and nerolidol may enhance exosome production and therapeutic efficacy through indirect modulation of mesenchymal stem cell signaling pathways. Previous studies have shown that these compounds regulate redox homeostasis, mitochondrial function, and inflammatory signaling cascades such as NF-κB and MAPK pathways processes that are increasingly recognized as key regulators of exosome biogenesis, secretion, and cargo composition. In this context, AA treatment may induce a metabolically activated yet phenotypically stable MSC state, thereby promoting increased exosome release and enhancing the anti-inflammatory and antioxidant properties of the resulting vesicles.

Several limitations of the present study should be acknowledged. the *in vitro* experiments were conducted using the C28/I2 immortalized human chondrocyte cell line rather than primary chondrocytes isolated from osteoarthritis patients. Although C28/I2 cells are widely used as a reproducible and well characterized model for mechanistic studies, they may not fully reflect the phenotypic heterogeneity, donor variability, and disease stage specific responses observed in primary human chondrocytes. Osteoarthritic conditions were simulated using H₂O₂-induced oxidative stress, which models a key pathological feature of OA but does not capture the full complexity of the joint microenvironment, including mechanical loading, immune cell interactions, and chronic cytokine exposure. This study did not include *in vivo* validation using animal models of osteoarthritis. As a result, important translational aspects such as exosome biodistribution, intra-articular retention, optimal dosing regimens, long-term safety, and therapeutic durability need to be evaluated.

**Figure 4:**
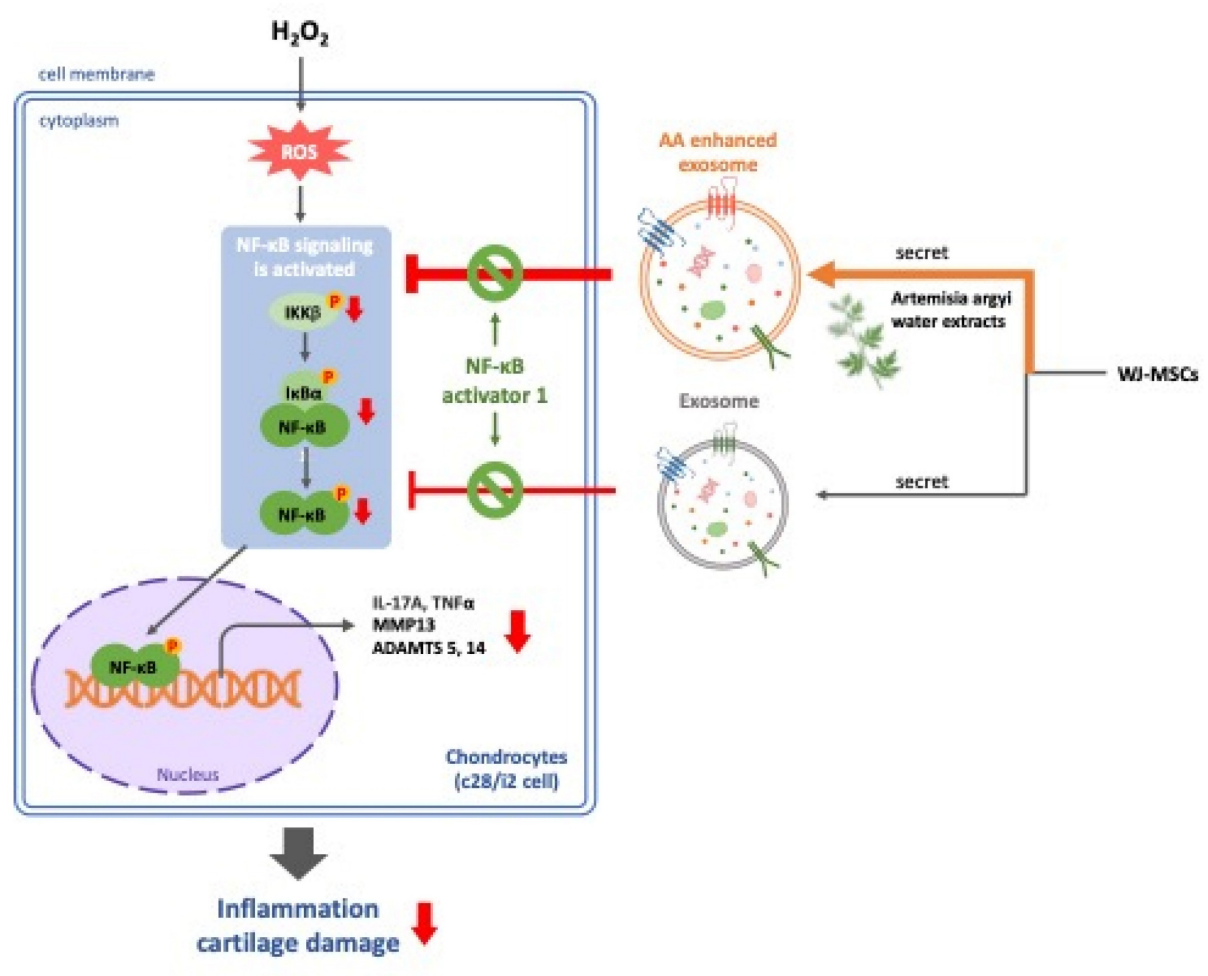
** AA-enhanced WJSCs-derived exosomes effectively alleviate H₂O₂-induced inflammation and cartilage damage through the inhibition of the NF-κB pathway, offering a promising therapeutic strategy for osteoarthritis by combining regenerative and anti-inflammatory effects**.

Based on the findings from our study, we conclude that AA-enhanced WJSCs -derived exosomes alleviate H₂O₂-induced inflammation and cartilage cell damage through the inhibition of the NF-κB signaling pathway (Figure 4). This suggests that AA has significant therapeutic potential, especially in protecting cartilage from inflammation-related joint diseases such as osteoarthritis. By combining the regenerative capabilities of MSC-derived exosomes with the anti-inflammatory and chondroprotective effects of AA, we propose a novel strategy for OA treatment that not only addresses symptom management but also targets the underlying mechanisms of cartilage degradation. This approach may offer a promising direction for more effective and sustainable therapies in OA patients.

## Figures and Tables

**Table 1:** Primer sequences

Gene name	Forward	Reverse
IL17A	AGATTACTACAACCGATCCACCT	GGGGACAGAGTTCATGTGGTA
TNF-alpha	ATGTGGCAAGAGATGGGGAA	CTCACACCCCACATCTGTCT
ADAMTS14	CCAATCGGAGGTTGGTAGTGC	CACACACTCCTGCCGTAAGG
ADAMTS5	ACTACGATGCAGCTATCCTGT	GTCCCAACGTCTGCCATTC
GAPDH	TGACCTTTCTGTAGCTGGGG	CAAGCCCACCCCTTCTCTAA

## Data Availability

All data generated or analyzed during this study are included in this article.

## Abbreviations

OA: Osteoarthritis
MSCs: mesenchymal stem cells
AA: *Artemisia Argyi*
WJSC: Wharton's jelly-derived mesenchymal stem cell
H_2_O_2_: hydrogen peroxide
ROS: reactive oxygen species
NF-κB: Nuclear factor kappa-light-chain-enhancer of activated B cells
GDF5: Growth Differentiation Factor 5
TNF-α: tumor necrosis factor-alpha
IL-1β: Interleukin-1 beta
IL-6: Interleukin-6
MMPs: Matrix metalloproteinases
SDF-1: stromal cell-derived factor 1
CXCR4: C-X-C Chemokine Receptor Type 4
PI3K: Phosphatidylinositol 3-kinase
Akt: Protein kinase B
TGF-β: Transforming growth factor beta
Smad3: Mothers against decapentaplegic homolog 3
NSAIDs: nonsteroidal anti-inflammatory drugs
TCM: Traditional Chinese Medicine
VEGF: Vascular Endothelial Growth Factor
ADAMTS14: A Disintegrin And Metalloproteinase with Thrombospondin Motifs 14
ADAMTS5: A Disintegrin And Metalloproteinase with Thrombospondin Motifs 5
Nanog: Nanog Homeobox
KLF4: Kruppel-Like Factor 4
CXCR7: C-X-C Chemokine Receptor Type
CD44: Cluster of Differentiation 44
CD90: Cluster of Differentiation 90
COL2A1: collagen type II alpha 1
MMP13: matrix metalloproteinase 13
